# Letters from Italy

**Published:** 1884-11

**Authors:** A. Lagorio

**Affiliations:** Chiavari, Italy


### CORRESPONDENCE.

LETTER FROM ITALY.

*Messrs. Editors:*—I take pleasure in sending for the benefit of your readers a brief report on cholera, which is everywhere about us. The cholera microbe, after bringing desolation, misery, and death to hundreds of peaceful homes in our neighboring cities of Toulon and Marseilles, has truly overleaped the quarantine, defied military cordons, and made its grand entrance upon our beautiful Riviera. Bravely have the Italian authorities fought against this germ, but in spite of their one hundred-ton Krupp guns, their two hundred and fifty thousand soldiers, their phenic acid, their chlorides, etc., they have lost the battle. Strict quarantines and plenty of fumigation have been of no avail; hundreds of our gay cities are infected by the epidemic; and even our beautiful Naples has succumbed; the last bulletin announcing to us the awful news that over five hundred were stricken down in this southern city alone, during the past twenty-four hours. What is it? What is the cause? What is to be done? is the general cry. A grand opportunity is offered for study and observation. The researches of Strauss and Roux (*Riv. Clin. e Terap.*) made in Toulon, have been a continuation of those instituted in Egypt last year with Thuiller and Nocard. From the first autopsy they have been enabled to confirm the identity of the lesions observed in the epidemic in Egypt. While there, Strauss and his collaborators attempted to find in the walls of the intestines a specific microbe. Their method consisted in coloring fragments of intestines, hardened in alcohol, with a solution of blue methylene. They saw in the superficial portions of the mucous membrane several micro-organisms, in variable number. The bacilli were numerous and variable, in aspect and dimensions. It was impossible for them to draw any positive conclusions, from these anatomical results, as to the cause of the disease. In Toulon they again attempted to find a specific organism in the intestinal walls. According to Koch, the cholera was characterized in Egypt, as in India, by the constant presence of a characteristic bacillus in the mucous membrane of the small intestines. The new observations of Strauss and Roux, in Toulon, confirm those observed in Egypt. In rapidly fatal cases the organisms were found in less quantity, and in some it was impossible to observe any. In some of his communications from Calcutta, Koch gives more accurate details of the organism, which he believes to be the cause of cholera; he describes the bacillus as not straight, but more or less curved, at times comma-shape, or semicircular. To show this microbe, Koch does not use any special process of coloring; he takes a small part of a dejection or of intestinal mucous, and places it on a slide; this preparation is slightly warmed, and colored with an aqueous concentrated solution of aniline,—he prefers it to the blue methylene.

The intestinal contents, taken from a cadaver in the best of conditions, show, in the greatest number of cases, various species of micro-organisms, among which is found the comma-bacillus. Koch has made several culture experiments with this bacillus. A small portion of the intestinal contents is diluted with a few cubic centimeters of gelatine tea; this is poured on a slide and allowed to cool and thicken. Among the developed organisms there are some presenting the aspect of small retracted pieces; they are formed by the comma-shaped organisms, which, under the microscope, seem to perform rapid movements. These organisms, during their vegetation, at times remain united by the ends, showing an S shape. Koch believes this subject of culture in the gelatine tea to be characteristic. The comma-bacillus needs an alkaline fluid for its development, and a temperature of 16° to 42° C.; a dessication of only a few hours destroys it. Now, can we believe this comma-shaped microbe to be the true organism of cholera? Not until we can produce the identical disease with the aid of these bacilli only. Koch supports his discovery by stating that this bacillus has never been found in a healthy body, nor in bodies dead from other diseases.

The members of Pasteur's Mission, in Egypt, discovered some extremely fine particles, suggesting an organism, in the blood of choleric patients. These are due to an alteration of the hæmoglobin.

According to Hayem, the following ought to be the therapeutics of cholera: For the premonitory diarrhœa, he advises opium; above all, hypodermic injections of morphine; the black sulphide of mercury, or the salicylate of bismuth; ice, etc., according to the case. During the stage of collapse there is only one indication: to replace the enormous loss of water and chloride of sodium ejected from the intestinal canal. He believes that there ought to be again experiments on a large scale, by the intravenous injections of distilled water, every 1000 grams containing 5 grams of chloride of sodium, 1 gram of hydrate of sodium, and 25 grams of sulphate of sodium.

This operation is called hypodermoclisis, and is at present largely tried in Naples, I am pleased to state, with the happiest results. The credit of this method of treatment is really due to Prof. Cantam, who describes it in his work on Therapeutics (Vol. I, page 115, 1867). His solution is as follows: 1000 grams of water, 4 grams of common salt, and 3 grams of carbonate of sodium. The temperature of the liquid to be injected ought to be that of the blood.

But very little being known positively as to the cause of cholera, Bouchut recommends a symptomatic treatment. If the patient be cold, he must be warmed; if he vomit, give the Riviere's potion, gaseous drinks, ice; if he have diarrhœa, give opium, astringents, acids, subnitrate of bismuth, etc. Those who believe that cholera is caused by special bacilli, use antiseptics, microbicides, and everything which is inspired by the parasitic theory of diseases. Ingeniously they believe that, the cholera poison being due to milliards and milliards of comma-bacilli in the intestines, it is necessary to give a few milligrams of the biniodide or bichloride of mercury; a few centigrams of sulphate of copper to destroy the cause, that is the parasite, and save the patient!

Before the invasion of cholera all this may be supported, but after the invasion, when the choleric fermentation has begun with all its alarming symptoms, it seems below the capacity of reasoning minds to pretend to be able to destroy the ferment in such a complex body as the human organism.

But as all diseases at present are zymotic and parasitic, our therapy is simplified and our materia medica limited to a notable degree. There is only one cause—the ferment or the zymosis; and one treatment—the anti-zymotic: mercury, iodine, carburets.

Besides this treatment of symptoms and that also of the intestinal poison producing the cholera, there is a third, which probably is not worth much more, it is that based upon the theory of the choleraic poisoning of the nervous system. When the cholera has progressed, there is noticed such a predominance of nervous accidents as to induce some to believe the disease to proceed under the influence of the pneumogastric nerve and the spinal medulla. The poisoning is rather cerebro-spinal than gastro-intestinal.

The uncontrollable vomiting is the effect of the pneumogastric trouble; the diarrhœa, the result of the sympathetic disturbance; and the cramps, a spinal phenomenon. The intestinal and gastric flux is but an effect of changes in the nervous supply of the intestines, and the bacilli there found are a consequence rather than a cause, and a *secondary phenomenon.*

The treatment, therefore, is not antiparasitic, but one which, acting on the cerebro-spinal system, can arrest the choleric discharge and its grave consequences. This treatment consists of hypodermic injections of morphine. Bouchut has used them in a young lady affected with cyanosis, cold, sunken eyes, vomiting, diarrhœa and albuminuria. Every evacuation was stopped with the injections, reaction was established and the cure was complete. In another case of sporadic cholera, only one injection given by Dr. Vattier was sufficient to decide the cure. This sort of treatment being rational, and with no risks, is highly recommended by Bouchut in the beginning of the disease; moreover, opium and laudanum when given internally may be vomited or not absorbed, while the injection of morphine is sure in its effects.

Trocin has made several experiments with the inhalations of oxygen in the Saint Mandrier Hospital in Toulon. In a number of cases the effects have been well marked; the pulse became stronger, the skin warmer, and the general aspect improved. These effects did not last long; and Dr. Rochard has not been able to announce any cures by this method of treatment. Further experiments ought to be instituted.

Luton urges hypodermic injections of a solution of a neutral salt, preferring the ordinary sulphate of sodium. The introduction of this solution in a organism exhausted from the excessive colliquative discharges, has the effect of a sanguineous transfusion. Moreover, a substance of this nature is the best agent for harmonizing the gastro-intestinal functions, causing the peristaltic movements to be antagonized by the anti-peristaltic. It reëstablishes diuresis; and eliminates harmful matter which circulated in the blood.

Much at present is written of Ethiop's mineral as a prophylactic and remedy for cholera; and many are the pretenders for the honor of its first suggestion. But the credit is due to Socrates Cadet, Professor of Physiology in the University of Rome. It has been used since 1837, with splendid results, during the successive epidemics at Rome, Comarco, Paris, in Russia and in Tunis. Wherever used, the cures are said to amount to 70 to 90 % of those attacked. As a prophylactic it is given to adults in daily doses of twenty centigrams with a little sugar. In cases of attack, the dose is a gram and twenty centigrams. If it be vomited, the dose is repeated; and after the first dose sixty centigrams are given hourly until every choleraic symptom has disappeared. A physician asserts that during an epidemic in India, he used this remedy only, and obtained 78 to 82 per cent. of cures, and 96 per cent. in those who had used it as a prophylactic. Innumerable remedies are daily recommended and warranted, but, alas! after a few trials they are relegated to oblivion!

At the first outbreak of the epidemic in Italy, the government made a earnest appeal to physicians to volunteer their work and skill in suppressing the disease and in treating its victims. This appeal had very little effect, for few responded; but through the presidents of the several medical societies of the State, our physicians assured the government that they were willing to risk their lives as sanitarians and citizens; but, before doing so, they asked for a law making them equal to officers of the army, so that if the government desired them to sacrifice their lives for the public health, it should assure them that their families would not have to suffer without receiving a suitable pension.

The government has not yet taken the matter in consideration, but it will be seriously discussed at the next Medical Congress, to be held in Turin about the end of this month. Our physicians, as to this question, are in the right.

A. LAGORIO, M. D.

Chiavari, Italy, Sept. 11th, 1884.

---

*To the Editors of the* CHICAGO MEDICAL JOURNAL AND EXAMINER:

*Messrs. Editors:* Many theories have been advanced by different authors on the essence of the febrile process, and the opinions of pathologists are yet much divided. The abnormal increase of temperature has been explained by a diminution in the dispersion of the animal heat (Traube); by an increase in the production of this heat; by a diminution in the dispersion, and increase in the production of heat existing contemporarily; and by extraordinary biochemical processes. (Murri).

The skin represents the principal and the most important regulating means of the animal heat, and the functional state of the cutaneous vessels, (on which depends the greater or less flow of blood to the periphery of the body,) is the one which regulates the dispersion of the heat.

All the different modern theories have recognized the alteration of the function of the cutaneous vessels to be the most important factor in the production of fever; by Traube it was considered as the only and the primary factor capable of directly producing an increase of temperature of the body; other pathologists, differing from Traube, admit it to be only a consequence of the abnormal temperature, therefore of secondary importance only.

In consequence of the uncertainties yet existing in the doctrine of the febrile process, our knowledge of the mechanism of the action of the antipyretics is uncertain. Every hypothesis so far given, explaining how apyrexia is produced artificially by internal remedies, have been reached by analogy, from the physiological properties of remedies, rather than from their action on the febrile process.

The new remedy, kairine, has been largely tried and studied in the clinic of Professor Maragliano in the Pammatone Hospital in Genoa, not only as a febrifuge in general, but also as producing its effects on the cutaneous vessels and circulation. The experiments have been conducted and recorded by the professor's assistant, Gueirolo, a bright student and an accurate observer. The movements of the sanguineous vessels were studied with the pletigmograph of Professor Mosso. The instrument was placed on the forearm and the thermometer in the armpit, for a time varying from four to eight hours. Kairine was given to different patients affected with intermittent fever, croupous pneumonia, and typhoid fever, in doses of a gram every hour for two or three hours, and he was enabled to prove that with the lowering of the febrile temperature with kairine there is a dilatation of the peripheral vessels; that this dilatation precedes for a while the lowering of the temperature. That as the action of the kairine is exhausted, the fever reappears, and there is a constriction of the peripheral vessels; that this constriction precedes for a while the increase of temperature, and is more rapid and energetic the more rapid is the rise of the temperature. These conclusions tend to prove that the constriction and the dilatation of the vessels are not secondary facts.

*Antipirine* is another new remedy largely used of late in this clinic as an antipyretic. It comes from Meister Lucius, of Frankfort-on-Màin, in the form of prismatic crystals, very soluble in water, and in alcohol, less in ether. Its solution has a neutral reaction. The experiments as to its action on fever were conducted by the assistant, Ampuguani. In a dose of 50 centigr. given at one time it did not have much effect; but in doses of one gram the depression commenced after an hour of its giving, and increased in five to six consecutive hours to 3 degrees. At a dose of 1½ gram the lowering was notable and more rapid. In repeated doses, the antipyretic effects of the alkaloid last six to eighteen hours, and sometimes thirty-six to forty-eight hours. Many phthisical patients remained without fever from one to two days.

There is no doubt that antipirine has marked antipyretic properties; although its action is less energetic than kairine yet it possesses the value to last a longer time.

I take pleasure in communicating for the benefit of your readers, a few observations of Prof. DeRenzi on the comparative effects of some antifebrile remedies used in his clinic. In the fever of phthisis he has used kairine, phenate of soda, salicylate of soda, the salts of quinine, phenic acid, resorcine, and benzoic acid. Of these, he found that kairine has the power of lowering the temperature quicker and stronger; but this lowering is not lasting. The phenate of soda lowers the temperature, as the salicylate of soda, for a longer time; is tolerated better; and does not produce such abundant sweats as kairine; but the greatest diminution of temperature obtained with the phenate of soda and the salicylate of soda is scarcely half of that obtained with kairine. The antifebrile power is more marked in the phenate than in the salicylate. The phenate is given in capsules containing 20 centigr. of the remedy. The dose of these is 6 to 16 a day. Of the salts of quinine, the arseniate has antipyretic effects superior to the sulphate. Benzoic acid and resorcin produced only slight lowering of temperature ; the phenic acid, none.

For the fever of pleurisy the remedies generally used are: kairine, salts of quinine, and the phenate of soda. Kairine does not have much of an action. Better than this are the salts of quinine the arseniates gave the most marked effect. The sodic phenate has been, in this clinic, the most energetic remedy. In a case of high fever, rebellious to other remedies, this salt reduced rapidly the temperature ; although kairine had been given at high doses (6 to 8 grams a day), and the sulph. of quinine (1.50 grams internally and ½ gram hypodermically), no change had been noticed. But the phenate of soda given for a few days (1 to 2.50 grams) entirely removed the fever.

Several antifebrile remedies have been urged in the treatment of typhoid fever and febriculæ. The cold pack always reduced the temperatures of 40 deg. centigr. to a normal state. In the febriculæ, quinine and salicylic acid were also used, but with little effect. For in almost all patients with typhoid fever the Professor adopted the carbolic acid spray at the bedside, in solution of 2 per cent., every hour. The efficacy of a carbolized atmosphere on the course of the fever was most evident. In several cases the fever was lowered ; in others, it seemed that this method of treatment abbreviated the disease.

In pneumonias of an adnamic type, alcohol is always used. While this remedy supports the patient, it has also shown an action on the temperature of the body. It was observed that in thirteen patients the temperature was kept lower than before the remedy was given. Quinine and kairine were given to lower rapidly and strongly the very high temperatures. The former, in doses of 2 grams, lowered the temperature, but always limited to about 1 degree; while kairine lowered the temperature 2 to 3 degrees. But this lowering with kairine is always transitory, the apyretic state lasting three to six hours.

For the malarial fevers, quinine, kairine and the electric current were used. The action of quinine has always been the surest and the most constant; kairine stopped definitely a fever of a quotidian type, in one case, in two days of treatment. The Faradic current showed a marked action.

The Faradic and galvanic currents were also used in two cases of symptomatic fever, of a catarrhal broncho-pneumonia, and of a pseudo-leukæmia, but with no effect whatever.

A. LAGORIO, M. D.

CHIAVARI, Italy, August 27, 1884.

